# Phase space dynamics of eye-movement deficits in psychiatric patients

**DOI:** 10.1192/j.eurpsy.2021.419

**Published:** 2021-08-13

**Authors:** S. Dal Col, R. Bolzani, M. Benassi, R. Sant’Angelo, R. Raggini, G. Piraccini

**Affiliations:** 1 Department Of Psychology, University of Bologna, Cesena, Italy; 2 Mental Health Department, AUSL ROMAGNA, Cesena, Italy

**Keywords:** inpatients, chaotic system analysis, space exploration, Fixational eye movements

## Abstract

**Introduction:**

Eye movement deficits in psychiatric patients have often been investigated with linear models, which fail to fully capture the complex dynamics characterizing eye movements.

**Objectives:**

The present work aims to investigate the deficits in fixational eye movements in psychiatric patients according as non-linear chaotic dynamic.

**Methods:**

We recruited 191 patients (91 males, average age 45 years) diagnosed with schizophrenia, bipolar disorder, depression and personality disorder. The control sample consisted of 22 healthy subjects (12 males, mean age 41 years). Fixational eye movements were recorded with the Eytribe infrared system and off-line analyzed using Matlab. The dynamics of fixation eye movements were investigated using a phase space graph, which refers to chaotic system analysis. This analysis allows to evaluate how the changes in space during fixation as a function of their speed.

**Results:**

A major difference emerged: psychiatric patients showed larger and faster eye movements gravitating around a single point of density, while control subjects exhibited slower and smaller eye movements with multiple drifts and microtremors.

**Conclusions:**

In conclusion, the dynamics of fixational eye movements in psychiatric patients seemed to be characterized by poorer efficiency in space exploration. These differences could be attributed to a worse coordination between the perceptual and the oculomotor system.

**Disclosure:**

No significant relationships.

